# The Effectiveness of Mind–Body Exercise on Health‐Related Quality of Life and Mental Health During and After Breast Cancer Treatment: An Umbrella Review of Meta‐Analyses for Randomized Controlled Trials

**DOI:** 10.1111/wvn.70008

**Published:** 2025-03-16

**Authors:** Jingsi Wen, Stanley Sai‐chuen Hui, Edwin Chun‐Yip Chin, Yijian Yang, Cindy Hui‐ping Sit

**Affiliations:** ^1^ Department of Sports Science and Physical Education The Chinese University of Hong Kong Shatin New Territories Hong Kong; ^2^ University of Macau Taipa Macau

**Keywords:** breast cancer, depression, mental health, mind–body therapies, qigong, quality of life, yoga

## Abstract

**Background:**

Previous studies regarding mind–body exercise among people with breast cancer mostly focused on one type of mind–body exercise and provided conflicting results.

**Aims:**

This paper aims to systematically synthesize the evidence hierarchy and examine the credibility of previous meta‐analyses of different types of mind–body exercises.

**Methods:**

We searched PubMed, Embase, Cochrane Library, Web of Science, and Epitemonikos from database inception to February 2nd, 2024, for meta‐analyses of randomized controlled trials. Included meta‐analyses examined the effects of mind–body exercises on at least one outcome of health‐related quality of life, cancer‐related fatigue, depression, anxiety, and sleep quality in breast cancer patients. The random effects estimates (Hedges'G), 95% prediction interval, small study effect, and excess significance bias were calculated. Furthermore, we categorized meta‐analyses based on the evidence credibility criteria and assessed quality using A Measurement Tool to Assess Systematic Reviews 2.

**Results:**

The umbrella review included a re‐analysis of 16 meta‐analyses of 9 articles including 134 randomized controlled trials involving 9469 breast cancer patients and survivors. We identified 3 articles as “low” quality and 6 articles as “critically low” quality. Convincing evidence supported the effectiveness of Yoga intervention in reducing depression symptoms (*G* = −0.77, 95% Confidence Interval [−0.93, −0.61]). However, 11 meta‐analyses were supported by weak evidence (1 for Qigong alleviated depression, 4 for Qigong, Baduanjin, Tai Chi Chuan, and Yoga improved health‐related quality of life, 3 for multiple mind–body exercises, Tai Chi Chuan, and Yoga reduced cancer‐related fatigue, 2 of Baduanjin and Yoga reduced anxiety, as well as 1 of Yoga improved sleep quality).

**Linking Evidence to Action:**

Mind–body exercises, especially yoga, may be beneficial for improving health‐related quality of life and mental health for breast cancer patients. Further high‐quality interventions investigating diverse mind–body exercise interventions are warranted to ascertain the effectiveness of health‐related quality of life and mental health outcomes.

## 1 Introduction

According to the latest Global Cancer Statistics, female breast cancer is the second leading cause of global cancer incidence in 2022 (Bray et al. 2024). A large proportion of breast cancer patients experience concurrent negative psychological and physical symptoms during their cancer care trajectory (Guimond et al. 2020). Rates of depression and anxiety among breast cancer patients range from 10% to 30% (Ng et al. 2017), and sleep disorder prevalence in breast cancer patients is estimated as high as 93% (Leysen et al. 2019). Approximately 1 in 4 women with breast cancer suffer from cancer‐related fatigue during and after treatment (Abrahams et al. 2016). These health challenges, especially mental disorders, may contribute to adverse events, including psychological illness, poor quality of life (QoL), and low treatment compliance (Elizabeth et al. 2023).

Complementary and integrative therapies are recommended by different clinical practice guidelines, especially for mental health and overall QoL during and after breast cancer treatment (Campbell et al. 2019; Carlson et al. 2023; Greenlee et al. 2017). Mind–body exercise stands out as a popular integrative approach among cancer patients and survivors (Lee et al. 2019) that integrates movement sequences, breathing control, and attention regulation (Blomstrand et al. 2023; Ye et al. 2022). It is low‐intensity, easy to practice, and emphasizes the connection between the brain, mind, and body (Geng et al. 2023).

Nowadays, many meta‐analyses of randomized controlled trials (RCTs) have examined the effects of different types of mind–body exercise on health‐related QoL (HRQoL) and mental health in breast cancer patients and survivors (Liu et al. 2020; Meng et al. 2021; Riley et al. 2011; Zhu et al. 2023). However, most of these meta‐analyses only focus on a single type of mind–body exercise, lacking a comprehensive synthesis or evaluation of this series of evidence. This umbrella review aimed to evaluate the effectiveness of mind–body exercise compared to other treatment options on HRQoL and mental health (cancer‐related fatigue, depression, anxiety, and sleep quality) among breast cancer patients and survivors.

## 2 Methods

### 2.1 Protocol and Registration

The protocol for this umbrella review was registered with PROSPERO (CRD42024507462), and the study strictly adhered to the Preferred Reporting Items for Systematic reviews and Meta‐Analyses guideline (Page et al. 2021).

### 2.2 Search Strategy

We searched PubMed, Embase, Cochrane Library, Web of Science, and Epitemonikos databases from the inception to 2nd February 2024 for meta‐analyses of RCTs. Search terms included “breast cancer,” “mind–body exercise,” “Tai Ji,” “Yoga,” “Qigong,” “Dance,” “Pilates,” “Baduanjin,” “Yijinjing,” “Wuqinxi,” “Liuzijue,” “quality of life,” “fatigue,” “depression,” “anxiety,” “sleep,” and “meta‐analysis.” Searches were not limited by language or publication date. The detailed search strategy for each database can be found in Table S1. Two authors (JW and SH) independently screened titles and abstracts, followed by full‐text examination of potentially eligible meta‐analyses. Any discrepancy in the literature screening between the two reviewers was resolved by a third reviewer (YY). A manual search of the reference lists of included reviews was performed in addition to the digital search to ensure that no relevant articles were missed.

### 2.3 Eligibility Criteria

Eligibility criteria were developed using the Participant, Intervention, Comparison, Outcome, and Study design (PICOS) framework. This umbrella review included meta‐analyses of RCTs (S) that examined the effectiveness of mind–body exercises (I) on HRQoL and mental health outcomes (O) in patients with breast cancer (P), compared to any other treatment options (C). The inclusion criteria for participants were breast cancer patients and survivors aged 18 years and above, without restrictions on stage or medical treatments (e.g., surgery, radiotherapy, and chemotherapy) received. The intervention could comprise solely mind–body exercise (Yoga, Pilates, dance, Tai Chi Chuan, Qigong, Baduanjin, Liuzijue, Wuqinxi, and Yijinjing) or mind–body exercise plus usual care/medical treatment, without restrictions on delivery methods (e.g., in‐person or online), duration, or frequency. There were no limitations on the comparator arm; it could be active exercise control (e.g., walking, stretching) or non‐active control (e.g., usual care, waitlist control, psychotherapy intervention). Meta‐analyses were required to report at least one of the following outcomes: HRQoL, cancer‐related fatigue, depression, anxiety, and sleep quality.

We excluded systematic reviews without meta‐analysis. We also excluded articles that did not provide adequate data for re‐analysis (e.g., lacking the information of sample size, mean, and standard deviation). To avoid overlaps, if more than one meta‐analysis on the same research question was available, we assessed only the study that included the largest number of primary studies. Meta‐analyses of fewer than three primary studies were also excluded.

### 2.4 Data Extraction

Two reviewers (JW and SH) independently extracted the following information from each eligible study: first author's surname, year of publication, search time, search databases, primary study designs, number of included primary studies, sample sizes, population, type of intervention and control, outcomes, measurements, and their quality assessment methods and results. For meta‐analyses reporting multiple outcomes, data were extracted separately for each outcome. For each outcome of different mind–body exercises, we respectively extracted the sample size, mean value, and standard deviation of the experimental group and control group for each primary study from the meta‐analysis. There was a meta‐analysis that included both RCTs and non‐RCTs; we only extracted the data from the RCTs for re‐analysis. In addition, only data from the immediate post‐intervention or the change between pre‐intervention and post‐intervention were considered, excluding the follow‐up data.

### 2.5 Quality Assessment of Included Meta‐Analyses

The methodological quality of the included meta‐analyses was independently assessed by two reviewers (JW and SH) using the AMSTAR‐2 checklist (i.e., A Measurement Tool to Assess Systematic Reviews 2), which comprises 16 items scored as yes, partial yes, or no (Supporting Information 1) (Shea et al. 2017). Seven items are considered “critical” and nine “non‐critical.” Meta‐analyses were rated as “high” (no or 1 non‐critical weakness), “moderate” (more than 1 non‐critical weakness), “low” (1 critical flaw with or without non‐critical weaknesses), or “critically low” (more than 1 critical flaw with or without non‐critical weaknesses) (Shea et al. 2017).

### 2.6 Statistical Analysis

We used individual study estimates to reanalyze each eligible meta‐analysis. For each outcome, we calculated the summary effect and the 95% confidence interval (95% CI) using the DerSimonian and Laird random‐effects model (DerSimonian and Laird 1986). Statistical significance was set at *p* < 0.05. All estimates in the original meta‐analysis were converted to Hedges'G to facilitate interpretation. To further account for heterogeneity between studies, we ran Cochran's Q test and calculated the I^2^ statistic (*I* ^2^ > 50% indicates high heterogeneity) (Higgins et al. 2003). Additionally, a 95% prediction interval was calculated to evaluate the uncertainty of effect size in new studies addressing the identical association (Riley et al. 2011). For the largest study of each meta‐analysis, we calculated the standard error of the effect size and 95% CI. The evidence for small study effects was assessed by the Egger regression asymmetry test, with *p* < 0.10 taken as statistical evidence of the presence of small study effects (Sterne et al. 2011). We also conducted an excess significance bias test, which evaluates whether the observed number of studies with statistically significant results differs from the expected number of positive studies, by using a χ^2^ test (Ioannidis and Trikalinos 2007). Excess significance bias was set at *p* ≤ 0.10.

Evidence from meta‐analyses was assessed in terms of the number of cases, statistical significance, heterogeneity, 95% prediction interval, largest study significance, small‐study effect, and excess significance. We evaluated the credibility of evidence regarding the effectiveness of each mind–body exercise intervention on each outcome that was categorized into five classes: convincing (Class I), highly suggestive (Class II), suggestive (Class III), weak (Class IV), and not significant (Class NS) (Ioannidis and Trikalinos 2007) (Table 1).

**TABLE 1:** Criteria used for the classification of the evidence.

Class	Criteria
Convincing (Class I)	Number of breast cancer cases > 1000 Random‐effects *p* < 1 × 10^−6^ *I* ^2^ < 50% 95% prediction interval excluding the null No small‐study effects No excess significance bias The largest study was concordant in terms of statistical significance with the random‐effects result
Highly suggestive (Class II)	Number of breast cancer cases > 1000 Random‐effects *p* < 1 × 10^−6^ Largest study with a statistically significant effect
Suggestive (Class III)	Number of breast cancer cases > 1000 Random‐effects *p* < 0.001
Weak (Class IV)	Random‐effects *p* < 0.05
Not significant (Class NS)	Random‐effects *p* > 0.05

Data analysis was conducted using Stata (version 17) and the Umbrella Review Package for R (version 4.3.3).

## 3 Results

**FIGURE 1:**
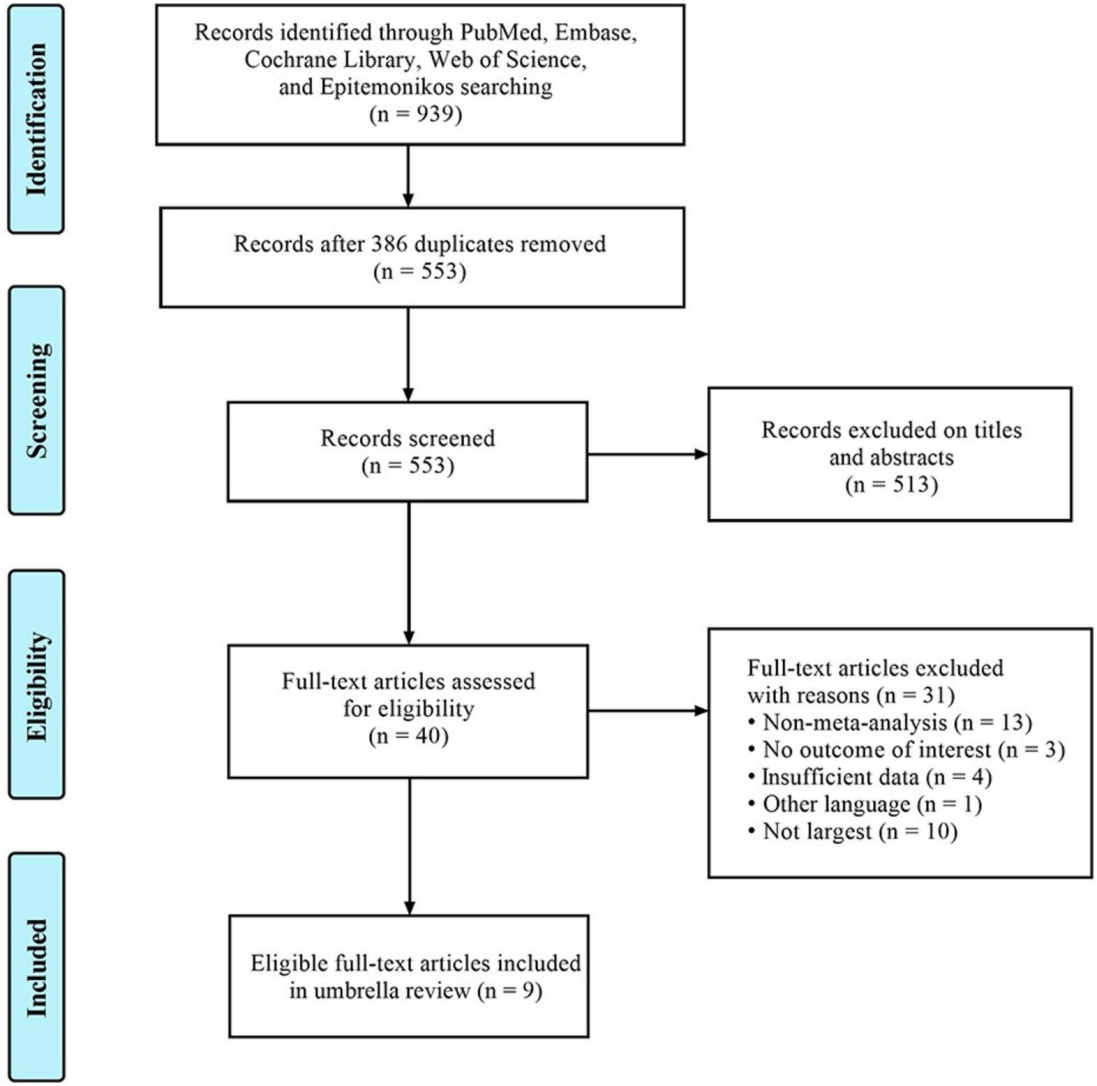
PRISMA flowchart.

A total of 939 publications were identified across 5 databases, with no additional meta‐analyses found through manually searching reference lists. After duplicate removal and inspection of titles and abstracts, 40 full‐text articles were assessed for eligibility. Details of the excluded articles and reasons are provided in Table S2. Finally, 9 articles were included to examine the effectiveness of mind–body exercise interventions on HRQoL and mental health in women with breast cancer (Figure 1).

### 3.1 Characteristics of Included Studies

Table S3 presents the characteristics of the included meta‐analyses, which were published between 2016 and 2023. The average number of search databases included in each meta‐analysis ranges from 5 to 8, with an average of 7 databases. Eight meta‐analyses included only RCTs (Cramer et al. 2017; Dong et al. 2019; Li et al. 2023; Liu et al. 2020; Liu et al. 2021; Ye et al. 2022; Zhu et al. 2023; Zuo et al. 2023). While 1 included both RCTs and non‐RCTs 2021, we included the RCTs data.

The average number of primary studies included in each meta‐analysis ranged from 7 to 24, with an average of 16 studies. All the participants included in the meta‐analyses were females with breast cancer, with the total number of participants ranging from 782 to 2166. To assess the methodological quality of each study, the Cochrane Risk‐of‐Bias tool (version 1) was used in 7 meta‐analyses, 12 Cochrane Back Review Group's risk of bias and Physiotherapy Evidence Database (i.e., PEDro) scale were used in the other two meta‐analyses. The details of the quality assessment of included meta‐analyses are provided in Table S3.

Five different types of mind–body exercise interventions were examined across the 9 meta‐analyses, including 4 Yoga interventions, 2 Tai Chi Chuan interventions, 1 Baduanjin intervention, 1 Qigong intervention, and multiple mind–body exercise interventions (including different primary studies of Yoga, Tai Chi Chuan, Qigong). The intervention duration ranged from 1 to 6 months, with frequencies ranging from 1 to 5 times per week and times (session durations) lasting from 20 to 90 min. The most common intervention for control groups was usual care; other control interventions included wait‐list control, psychotherapy, and active exercise control (e.g., walking, stretching).

Outcomes measures encompassed HRQoL, cancer‐related fatigue, depression, anxiety, and sleep quality, assessed through self‐reported or clinician‐rated questionnaires. The most frequently used measurement tools included the Functional Assessment of Cancer Therapy–Breast, Fatigue Symptom Inventory, Center for Epidemiological Studies Depression Scale, Self‐rating Anxiety Scale, and Pittsburgh Sleep Quality Index. After applying the inclusion criteria, 9 articles and 16 distinct meta‐analyses were included in this umbrella review.

### 3.2 Methodological Quality Assessment

The evaluation of methodological quality using AMSTAR‐2 revealed that 3 (33.3%) meta‐analyses were regarded as “low” quality, while 6 (66.7%) were rated as “critically low” quality (Tables S4 and S5). The most common critical flaw was “did not report the protocol registration” (*n* = 6). For justification for excluding individual studies, 5 meta‐analyses graded “partial yes,” which provided a list of all potentially relevant studies that were read in full‐text form but excluded from the review, and 1 meta‐analysis did not provide any list of excluded studies. Four meta‐analyses did not consider the impact of risk of bias during interpretation, and 4 meta‐analyses did not consider publication bias. All meta‐analyses used appropriate methods for literature search, quality assessment, and data analysis.

No studies reported on sources of funding for articles included in their meta‐analyses (*n* = 9). Only 4 meta‐analyses graded “yes” in describing the included studies in adequate detail, and the other 5 meta‐analyses graded “partial yes.” For data extraction in duplicate and the impact of risk of bias in individual studies on the results, only 1 meta‐analysis failed to meet the criteria. All meta‐analyses met the other five non‐critical items criteria for reporting PICO, explanation of the selection of the study designs, performing study selection in duplicate, explanation for any heterogeneity observed in the results, and any potential sources of conflict of interest.

### 3.3 Summary Effect Size

**FIGURE 2:**
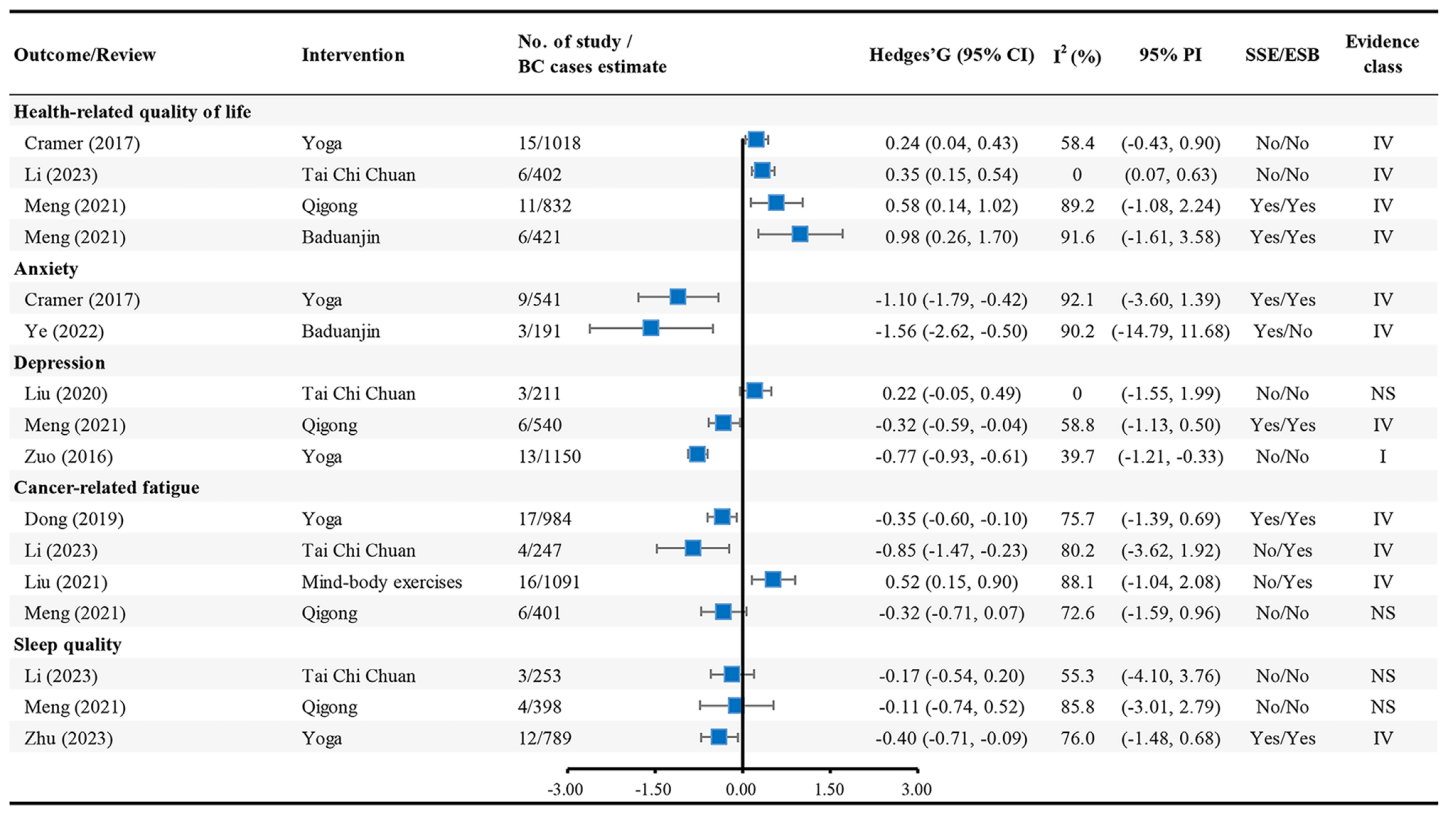
Forest plot of health‐related quality of life and mental health outcomes.

In the included 9 eligible articles and 16 distinct meta‐analyses, only 3 (18.75%) meta‐analyses had over 1000 breast cancer cases. At a threshold of *p* < 0.05, the summary random effects estimates were significant in 12 of the 16 (75%) meta‐analyses (Table S5 and Figure 2). At a more conservative threshold of *p* < 0.001, 2 (12.5%) estimates were significant in random effects models. At a threshold of *p* < 10^−6^, only 1 (6.25%) meta‐analysis was statistically significant in the random effects model, which found that Yoga intervention could significantly decrease depression symptoms among breast cancer patients.

### 3.4 Heterogeneity Between Studies

Thirteen (81.25%) meta‐analyses showed high heterogeneity (*I* ^2^ > 50%). Only 3 (18.75%) meta‐analyses had little or no evidence of heterogeneity (*I* ^2^ < 50%) (Table S5 and Figure 2). We further assessed the uncertainty of the summary effects by calculating their 95% prediction intervals; however, the null value was excluded in 2 meta‐analyses (Yoga for depression and Tai Chi Chuan for HRQoL).

### 3.5 Small Study Effects and Excess Significance

There was no evidence for the presence of small study effects according to Egger's test in 9 (56.25%) meta‐analyses (Table S5 and Figure 2), including Yoga for HRQoL and depression; Qigong for cancer‐related fatigue and sleep quality; Tai Chi Chuan for HRQoL, cancer‐related fatigue, depression, and sleep quality; and mind–body exercises for cancer‐related fatigue.

No evidence of excess significance bias was found in 8 (50%) meta‐analyses, namely Yoga for HRQoL and depression; Qigong for cancer‐related fatigue and sleep quality; Baduanjin for anxiety; Tai Chi Chuan for HRQoL, depression, and sleep quality.

### 3.6 Grading the Evidence

We explored whether the reported meta‐analyses of different mind–body exercises were supported by convincing, highly suggestive, suggestive, weak, or not significant evidence. Only 1 (6.25%) meta‐analysis was supported by convincing evidence, which was Yoga for reducing depression symptoms. There was no meta‐analysis supported by highly suggestive or suggestive evidence, while 11 (68.75%) meta‐analyses were supported by weak evidence, including Qigong for depression; Qigong, Baduanjin, Tai Chi Chuan, and Yoga for HRQoL; mind–body exercises, Tai Chi Chuan, and Yoga for cancer‐related fatigue; Baduanjin and Yoga for anxiety; as well as Yoga for sleep quality. Finally, the remaining 4 (25%) meta‐analyses were supported by not significant evidence, namely Tai Chi Chuan for depression; Qigong for cancer‐related fatigue; Qigong and Tai Chi Chuan for sleep quality. Details of evidence grading are provided in Table S5 and Figure 2.

## 4 Discussion

To the best of our knowledge, this is the first umbrella review investigating the effectiveness of mind–body exercise on HRQoL and mental health in breast cancer patients and survivors. Firstly, the methodological quality of the included articles was low and critically low. Secondly, our analyses indicated that the positive effect of yoga on relieving depressive symptoms was graded convincing (class I). Thirdly, the effect of other mind–body exercises on other outcomes was graded weak (class IV) or not significant (class NS).

Our findings indicate solid evidence that all mind–body exercises included in this umbrella review can improve the HRQoL in breast cancer patients and survivors (Cramer et al. 2017; Li et al. 2023; Meng et al. 2021). Different HRQoL questionnaires may lead to inconsistent conclusions across studies (Banasik et al. 2011; Oh et al. 2008). Given that different QoL questionnaires focus on different aspects, it is essential to establish a standard QoL evaluation system for breast cancer women (Mokhtari‐Hessari and Montazeri 2020).

The American Society of Clinical Oncology guidelines recommend Yoga to relieve anxiety and depression symptoms during and after breast cancer treatment, and Tai Chi Chuan and Qigong are only recommended post‐treatment (Carlson et al. 2023). Weak evidence was observed in Yoga and Baduanjin for anxiety symptoms among people with breast cancer. Our study only included these 2 types of mind–body exercises for anxiety, and the sample size was relatively small. Furthermore, we found that Yoga could significantly reduce depression symptoms in women with breast cancer, supported by convincing evidence (Zuo et al. 2016), whereas Qigong and Tai Chi Chuan are supported by weak or not significant evidence. Collectively, most of these RCTs were conducted with a small sample size in a single center and focused on Chinese participants. Hence, there is a pressing need for more high‐quality trials to comprehensively explore the effect of different types of mind–body exercises on anxiety and depression symptoms in people with breast cancer.

Our study shows that multiple mind–body exercises, Yoga, and Tai Chi Chuan can significantly reduce cancer‐related fatigue scores (Dong et al. 2019; Li et al. 2023; Liu et al. 2021). Cancer‐related fatigue symptoms may increase proinflammatory factors, and mind–body exercises could reduce cancer‐related fatigue symptoms in people with breast cancer by reducing the inflammatory cytokines (Gould et al. 2013; Liu et al. 2021). However, our results did not find significant evidence of Qigong for reducing cancer‐related fatigue (Meng et al. 2021). Qigong interventions consisted of different modalities (e.g., Baduanjin, Chan‐Chuang Qigong, Guolin Qigong), duration (15 to 60 min per day), frequency (2 to 7 times per week), and time (2 to 6 months) (Meng et al. 2021), which may affect the assessment of the effects of Qigong, as well as the credibility rating of the evidence.

Our study revealed that among different mind–body exercises, Yoga emerged as the only intervention that significantly improved the sleep quality of breast cancer patients (Zhu et al. 2023). This positive effect can be attributed to the ability of mind–body exercises to reduce sympathetic arousal and inflammation, both of which play a role in influencing sleep quality in this population (Black et al. 2014; Manconi et al. 2010). Contrary to our findings, the effectiveness of Tai Chi Chuan and Qigong in improving sleep quality among breast cancer patients was not statistically significant (Irwin et al. 2017; Liu et al. 2015). Our analysis included 7 RCTs focusing on these mind–body exercise interventions, 5 of which specifically targeted post‐treatment breast cancer survivors with high baseline sleep quality scores. Consequently, these individuals did not demonstrate a significant improvement in sleep quality following the interventions.

There are several limitations in this study. First, there was a high degree of heterogeneity among the included articles in terms of the interventions utilized and outcome measurements assessed. Additionally, less common mind–body exercises, such as Pilates, dance, and traditional Chinese exercises, were underrepresented in the literature. For the same type of mind–body exercise, we could not consider the details of the intervention (duration, frequency, time, and delivery method), making it challenging to determine which aspects were most beneficial. Second, the methodological quality for most of the included meta‐analyses was rated as critically low, with no studies reaching a high or moderate level of quality. This suggests that the overall rigor and reliability of the studies included in this analysis may be compromised. Third, it is important to note that the studies included in this umbrella review were limited to single‐center studies and failed to adequately address the potential biases.

## Conflicts of Interest

The authors declare no conflicts of interest.

## Linking Evidence to Action

- Mind–body exercises, especially yoga, had beneficial effects on health‐related quality of life and most mental health outcomes during and after breast cancer treatment.
- Further high‐quality interventions exploring diverse mind–body exercise forms are needed to confirm the effectiveness of health‐related quality of life and mental health outcomes.
- Future studies could conduct a larger sample size, focus more on the less common mind–body exercise, and minimize the heterogeneity of intervention regimes and measurement tools.
- This study can serve as a specific guide to mind–body exercise interventions for caregivers assisting in breast cancer recovery and mental health management.

## Supporting information


Data S1.

## Data Availability

The data that supports the findings of this study are available in the Supporting Information of this article.
