# Supplementary material for: The Effectiveness of Mind–Body Exercise on Health‐Related Quality of Life and Mental Health During and After Breast Cancer Treatment: An Umbrella Review of Meta‐Analyses for Randomized Controlled Trials
Source: Worldviews Evid Based Nurs. 2025 Mar 16;22(2):e70008. doi: 10.1111/wvn.70008 (PMC11911294; doi:10.1111/wvn.70008)
Supplement: Supplementary file 1 — Data S1. [file WVN-22-0-s001.docx]

**Supplementary appendix**

**Contents**

[Supplementary Material 1. AMSTAR-2 items detail guideline 2](#_Toc183164125)

[Table S1: Detailed Search strategy for all databases 4](#_Toc183164126)

[Table S2: List of excluded studies, with reasons for exclusion after full-text reading 6](#_Toc183164127)

[Table S3. Characteristics of included meta-analyses. 8](#_Toc183164128)

[Table S4. AMSTAR-2 rating. 11](#_Toc183164129)

[Table S5. Quantitative synthesis and evidence grading criteria of the 16 meta-analyses comparing mind-body exercise interventions and controls. 12](#_Toc183164130)

#

# Supplementary Material 1. AMSTAR-2 items detail guideline

AMSTAR2 questions (adapted from Shea et al. 2017, Berendsen et al. 2024):

1. Did the research questions and inclusion criteria for the review include the components of PICO?

For Yes: appraisers should be confident that the four elements of PICO are described somewhere in the report.

For No: any element of the PICO is missing in the report.

2. Did the report of the review contain an explicit statement that the review methods were established prior to the conduct of the review and did the report justify any significant deviations from the protocol?
For Partial Yes: The authors state that they had a written protocol or guide that included all the following: review question(s), a search strategy, inclusion/exclusion criteria, and a risk of bias assessment.

For Yes: authors should demonstrate that they have worked with a written protocol. In addition, the protocol should be registered and should also have specified: a meta-analysis/synthesis plan, if appropriate, and, a plan for investigating causes of heterogeneity, justification for any

deviations from the protocol.

For No: if any of the above-mentioned elements are missing.

3. Did the review authors explain their selection of the study designs for inclusion in the review?

For Yes: the review should satisfy one of the following elements: an explanation for including only randomized controlled trials (RCTs) or only NRSI or both RCTs and NRSI.

For No: if the abovementioned elements are missing.

4. Did the review authors use a comprehensive literature search strategy?

Partial Yes: all the following should be completed: searched at least 2 databases (relevant to research question), provided keyword and/or search strategy, justified publication restrictions (e.g. language).

For Yes: in addition to all elements of partial yes, all the following has to be completed too: searched the reference lists/bibliographies of included studies. searched trial/study registries, included/consulted content experts in the field, if relevant, searched for grey literature, conducted search within 24 months of completion of the review.

For No: the abovementioned is missing.

5. Did the review authors perform study selection in duplicate?

For Yes: either one of the following: at least two reviewers independently agreed on selection of eligible studies and achieved consensus on which studies to include; or two reviewers selected a sample of eligible studies and achieved good agreement (at least 80 percent), with the remainder selected by one reviewer.

For No: the abovementioned is missing.

6. Did the review authors perform data extraction in duplicate?

For Yes: either one of the following: at least two reviewers achieved consensus on which data to extract from included studies, or two reviewers extracted data from a sample of eligible studies and achieved good agreement (at least 80 percent), with the remainder extracted by one reviewer.

For No: the abovementioned elements are missing.

7. Did the review authors provide a list of excluded studies and justify the exclusions?

For Partial Yes: provide a list of all potentially relevant studies that were read in full-text form but excluded from the review.

For Yes: must also have justified the exclusion from the review of each potentially relevant study.

For No: the abovementioned elements are missing.

8. Did the review authors describe the included studies in adequate detail?

For Partial Yes (all the following): described populations, interventions, comparators, outcomes, research designs.

For Yes (have all the following): described population in detail, intervention and comparator in detail (including doses if relevant, study’s setting, and timeframe for follow-up.

For No: the abovementioned elements are missing.

9. Did the review authors use a satisfactory technique for assessing the risk of bias (RoB) in individual studies that were included in the review? [RCTs]

For Partial Yes: must have assessed RoB from unconcealed allocation, and lack of blinding of patients and assessors when assessing outcomes (unnecessary for objective outcomes such as all-cause mortality).

For Yes: must also have assessed RoB from allocation sequence that was not truly random, and selection of the reported result from among multiple measurements or analyses of a specified outcome.

For No: the abovementioned elements are missing.

10. Did the review authors report on the sources of funding for the studies included in the review?

For Yes: must have reported on the sources of funding for individual studies included in the review. Note: Reporting that the reviewers looked for this information but it was not reported by study authors also qualifies

For No: the abovementioned elements are missing.

11. If meta-analysis was performed did the review authors use appropriate methods for statistical combination of results? [RCTs]

For Yes: the authors justified combining the data in a meta-analysis and, they used an appropriate weighted technique to combine study results and adjusted for heterogeneity if present. And investigated the causes of any heterogeneity.

For No: the abovementioned elements are missing.

12. If meta-analysis was performed, did the review authors assess the potential impact of RoB in individual studies on the results of the meta-analysis or other evidence synthesis?

For Yes: included only low risk of bias RCTs, or if the pooled estimate was based on RCTs at variable RoB, the authors performed analyses to investigate possible impact of RoB on summary estimates of effect.

For no: the abovementioned elements are missing.

13. Did the review authors account for RoB in individual studies when interpreting/discussing the results of the review?

For Yes: included only low risk of bias RCTs, or if RCTs with moderate or high RoB, and provided a discussion of the likely impact of RoB on the results.

For No: the abovementioned elements are missing.

14. Did the review authors provide a satisfactory explanation for, and discussion of, any heterogeneity observed in the results of the review?

For Yes: there was no significant heterogeneity in the results, or if heterogeneity was present the authors performed an investigation of sources of any heterogeneity in the results and discussed the impact of this on the results of the review.

For No: the abovementioned elements are missing.

15. If they performed quantitative synthesis did the review authors carry out an adequate investigation of publication bias (small study bias) and discuss its likely impact on the results of the review?

For Yes: performed graphical or statistical tests for publication bias and discussed the likelihood and magnitude of impact of publication bias.

For No: the abovementioned elements are missing.

16. Did the review authors report any potential sources of conflict of interest, including any funding they received for conducting the review?

For Yes: the authors reported no competing interests or the authors described their funding sources and how they managed potential conflicts of interest.

For No: the abovementioned elements are missin

# Table S1: Detailed Search strategy for all databases

| **Database** | **Search Term** | **Result** |
| --- | --- | --- |
| **PubMed** | #1: "breast neoplasms"[MeSH Terms] OR ("breast"[All Fields] AND "neoplasms"[All Fields]) OR "breast neoplasms"[All Fields] OR ("breast"[All Fields] AND "neoplasm"[All Fields]) OR "breast neoplasm"[All Fields] OR ("breast neoplasms"[MeSH Terms] OR ("breast"[All Fields] AND "neoplasms"[All Fields]) OR "breast neoplasms"[All Fields] OR ("breast"[All Fields] AND "tumor"[All Fields]) OR "breast tumor"[All Fields]) OR ("breast neoplasms"[MeSH Terms] OR ("breast"[All Fields] AND "neoplasms"[All Fields]) OR "breast neoplasms"[All Fields] OR ("breast"[All Fields] AND "cancer"[All Fields]) OR "breast cancer"[All Fields]) OR ("breast neoplasms"[MeSH Terms] OR ("breast"[All Fields] AND "neoplasms"[All Fields]) OR "breast neoplasms"[All Fields] OR ("breast"[All Fields] AND "carcinoma"[All Fields]) OR "breast carcinoma"[All Fields]) | 519321 |
|  | #2: ("Mind-body"[All Fields] AND ("exercise"[MeSH Terms] OR "exercise"[All Fields] OR "exercises"[All Fields] OR "exercise therapy"[MeSH Terms] OR ("exercise"[All Fields] AND "therapy"[All Fields]) OR "exercise therapy"[All Fields] OR "exercising"[All Fields] OR "exercise s"[All Fields] OR "exercised"[All Fields] OR "exerciser"[All Fields] OR "exercisers"[All Fields])) OR ("Mind-body"[All Fields] AND ("intervention s"[All Fields] OR "interventions"[All Fields] OR "interventive"[All Fields] OR "methods"[MeSH Terms] OR "methods"[All Fields] OR "intervention"[All Fields] OR "interventional"[All Fields])) OR ("Mind-body"[All Fields] AND ("movement"[MeSH Terms] OR "movement"[All Fields] OR "movements"[All Fields] OR "movement s"[All Fields])) OR ("mind body therapies"[MeSH Terms] OR ("Mind-body"[All Fields] AND "therapies"[All Fields]) OR "mind body therapies"[All Fields] OR ("mind"[All Fields] AND "body"[All Fields] AND "therapy"[All Fields]) OR "mind body therapy"[All Fields]) OR ("tai ji"[MeSH Terms] OR ("tai"[All Fields] AND "ji"[All Fields]) OR "tai ji"[All Fields]) OR ("tai ji"[MeSH Terms] OR ("tai"[All Fields] AND "ji"[All Fields]) OR "tai ji"[All Fields] OR "taiji"[All Fields]) OR ("tai ji"[MeSH Terms] OR ("tai"[All Fields] AND "ji"[All Fields]) OR "tai ji"[All Fields] OR ("tai"[All Fields] AND "chi"[All Fields]) OR "tai chi"[All Fields]) OR ("tai ji"[MeSH Terms] OR ("tai"[All Fields] AND "ji"[All Fields]) OR "tai ji"[All Fields] OR "taijiquan"[All Fields]) OR ("tai ji"[MeSH Terms] OR ("tai"[All Fields] AND "ji"[All Fields]) OR "tai ji"[All Fields] OR ("tai"[All Fields] AND "chi"[All Fields] AND "chuan"[All Fields]) OR "tai chi chuan"[All Fields]) OR ("yoga"[MeSH Terms] OR "yoga"[All Fields]) OR ("qigong"[MeSH Terms] OR "qigong"[All Fields]) OR ("danced"[All Fields] OR "dancing"[MeSH Terms] OR "dancing"[All Fields] OR "dance"[All Fields] OR "dances"[All Fields]) OR ("pilate"[All Fields] OR "pilates"[All Fields]) OR "Baduanjin"[All Fields] OR "Yijinjing"[All Fields] OR "Wuqinxi"[All Fields] OR "Liuzijue"[All Fields] | 70831 |
|  | #3："meta analysis"[Publication Type] OR "meta analysis as topic"[MeSH Terms] OR "meta analysis"[All Fields] OR (("meta"[Journal] OR "meta"[All Fields]) AND ("metabolism"[MeSH Terms] OR "metabolism"[All Fields] OR "synthesis"[All Fields])) OR ("meta analysis"[Publication Type] OR "meta analysis as topic"[MeSH Terms] OR "meta analyses"[All Fields]) OR (("classification"[MeSH Terms] OR "classification"[All Fields] OR "systematic"[All Fields] OR "classification"[MeSH Subheading] OR "systematics"[All Fields] OR "systematical"[All Fields] OR "systematically"[All Fields] OR "systematisation"[All Fields] OR "systematise"[All Fields] OR "systematised"[All Fields] OR "systematization"[All Fields] OR "systematizations"[All Fields] OR "systematize"[All Fields] OR "systematized"[All Fields] OR "systematizes"[All Fields] OR "systematizing"[All Fields]) AND ("review"[Publication Type] OR "review literature as topic"[MeSH Terms] OR "literature review"[All Fields])) OR ("systematic review"[Publication Type] OR "systematic reviews as topic"[MeSH Terms] OR "systematic review"[All Fields]) | 679001 |
|  | #4: #1 AND #2 AND #3 | **170** |
| **Web of Science** | #1: TS=(Breast Neoplasm OR Breast Tumor OR Breast Cancer OR Breast Carcinoma) | 741215 |
|  | #2: TS=(Mind-body exercise OR Mind-body intervention OR Mind-body movement OR Mind-body therapy OR Tai Ji OR Taiji OR Tai Chi OR Taijiquan OR Tai Chi Chuan OR Yoga OR Qigong OR Dance OR Pilates OR Baduanjin OR Yijinjing OR Wuqinxi OR Liuzijue) | 77409 |
|  | #3: TS=(meta-analysis OR meta synthesis OR meta-analyses OR systematic literature review OR systematic review) | 630847 |
|  | #4: #1 AND #2 AND #3 | **279** |
| **Ovid EMBASE** | #1: (Breast Neoplasm or Breast Tumor or Breast Cancer or Breast Carcinoma).mp. [mp=title, abstract, heading word, drug trade name, original title, device manufacturer, drug manufacturer, device trade name, keyword heading word, floating subheading word, candidate term word] | 731455 |
|  | #2: (Mind-body exercise or Mind-body intervention or Mind-body movement or Mind-body therapy or Tai Ji or Taiji or Tai Chi or Taijiquan or Tai Chi Chuan or Yoga or Qigong or Dance or Pilates or Baduanjin or Yijinjing or Wuqinxi or Liuzijue).mp. [mp=title, abstract, heading word, drug trade name, original title, device manufacturer, drug manufacturer, device trade name, keyword heading word, floating subheading word, candidate term word] | 28293 |
|  | #3: (meta-analysis or meta synthesis or meta-analyses or systematic literature review or systematic review).mp. [mp=title, abstract, heading word, drug trade name, original title, device manufacturer, drug manufacturer, device trade name, keyword heading word, floating subheading word, candidate term word] | 759505 |
|  | #4: #1 AND #2 AND #3 | **262** |
| **Cochrane Library** | #1: (Breast Neoplasm OR Breast Tumor OR Breast Cancer OR Breast Carcinoma) (Word variations have been searched) | 48303 |
|  | #2: (Mind-body exercise OR Mind-body intervention OR Mind-body movement OR Mind-body therapy OR Tai Ji OR Taiji OR Tai Chi OR Taijiquan OR Tai Chi Chuan OR Yoga OR Qigong OR Dance OR Pilates OR Baduanjin OR Yijinjing OR Wuqinxi OR Liuzijue) (Word variations have been searched) | 12503 |
|  | #3: (meta-analysis OR meta synthesis OR meta-analyses OR systematic literature review OR systematic review) (Word variations have been searched) | 54742 |
|  | #4: #1 AND #2 AND #3 | **115** |
| **Epistemonikos** | #1: (title:(Breast Neoplasm) OR abstract:(Breast Neoplasm)) OR (title:(Breast Tumor) OR abstract:(Breast Tumor)) OR (title:(Breast Cancer) OR abstract:(Breast Cancer)) OR (title:(Breast Carcinoma) OR abstract:(Breast Carcinoma)) | 75632 |
|  | #2: (title:(Mind-body exercise) OR abstract:(Mind-body exercise)) OR (title:(Mind-body intervention) OR abstract:(Mind-body intervention)) OR (title:(Mind-body movement) OR abstract:(Mind-body movement)) OR (title:(Mind-body therapy) OR abstract:(Mind-body therapy)) OR (title:(Tai Ji) OR abstract:(Tai Ji)) OR (title:(Taiji) OR abstract:(Taiji)) OR (title:(Tai Chi Chuan) OR abstract:(Tai Chi Chuan)) OR (title:(Yoga) OR abstract:(Yoga)) OR (title:(Qigong) OR abstract:(Qigong)) OR (title:(Dance) OR abstract:(Dance)) OR (title:(Pilates) OR abstract:(Pilates)) OR (title:(Baduanjin) OR abstract:(Baduanjin)) OR (title:(Yijinjing) OR abstract:(Yijinjing)) OR (title:(Wuqinxi) OR abstract:(Wuqinxi)) OR (title:(Liuzijue) OR abstract:(Liuzijue)) | 7119 |
|  | #3: (title:(meta-analysis) OR abstract:(meta-analysis)) OR (title:(meta synthesis) OR abstract:(meta synthesis)) OR (title:(meta-analyses) OR abstract:(meta-analyses)) OR (title:(systematic literature review) OR abstract:(systematic literature review)) OR (title:(systematic review) OR abstract:(systematic review)) | 455904 |
|  | #4: #1 AND #2 AND #3 | **113** |
| **Total** | **939** | |
| **Time** | **2 February 2024** | |

# Table S2: List of excluded studies, with reasons for exclusion after full-text reading

| **Author (year)** | **Title** | **Reason for exclusion** |
| --- | --- | --- |
| Cramer et al (2012) | Can yoga improve fatigue in breast cancer patients? A systematic review | Letter to the editor, not a meta-analysis. |
| Harder et al (2012) | Randomised controlled trials of yoga interventions for women with breast cancer: A systematic literature review | A systematic review, not a meta-analysis. |
| Srivastava et al (2015) | Effect of yoga on post-chemotherapy cognitive impairment in breast cancer patients: A systematic review | Conference abstract, not a meta-analysis. |
| Srivastava et al (2016) | Effect of yoga on the post chemotherapy cognitive impairment in breast cancer patients: A systematic review | Conference abstract, not a meta-analysis. |
| Sharma et al (2016) | A systematic review of yoga interventions as integrative treatment in breast cancer | A systematic review, not a meta-analysis. |
| Galliford et al (2017) | Salute to the sun: a new dawn in yoga therapy for breast cancer | A systematic review, not a meta-analysis. |
| Wei et al (2019) | Effectiveness of Yoga Interventions in Breast Cancer-Related lymphedema: A systematic review | A systematic review, not a meta-analysis. |
| Fatkulina et al (2021) | Dance/Movement Therapy as an Intervention in Breast Cancer Patients: A Systematic Review | A systematic review, not a meta-analysis. |
| Saraswathi et al (2021) | Managing Lymphedema, Increasing Range of Motion, and Quality of Life through Yoga Therapy among Breast Cancer Survivors: A Systematic Review | A systematic review, not a meta-analysis. |
| Selvan et al (2022) | Systematic review of yoga for symptom management during conventional treatment of breast cancer patients | A systematic review, not a meta-analysis. |
| Levenhagen et al (2023) | Effect of Yoga among Women at Risk and with Breast Cancer-Related Lymphedema: A Systematic Review | A systematic review, not a meta-analysis. |
| Nair et al (2023) | Assessment of the Impact of Yoga on the Quality of Life of Breast Cancer Patients: A Systematic Literature Review | A systematic review, not a meta-analysis. |
| Geng et al (2023) | Comparative efficacy of mind-body exercise for depression in breast cancer survivors: A systematic review and network meta-analysis | A network meta-analysis. |
| Espindula et al (2017) | Pilates for breast cancer: A systematic review and meta-analysis | Outcome not interest. Only analyzed functional capacity. |
| Wanchai et al (2020) | The effects of yoga on breast-cancer-related lymphedema: a systematic review | Outcome not interest. Only analyzed breast-cancer-related lymphedema. |
| Kaje et al (2023) | Effect of Yoga Intervention on Inflammatory Biomarkers among Women with Breast Cancer - A Systematic Review | Outcome not interest. Only analyzed inflammatory biomarkers. |
| Pinto-Carral et al (2018) | Pilates for women with breast cancer: A systematic review and meta-analysis | No sufficient data (mean and SD) for re-analysis. |
| El-Hashimi et al (2019) | Yoga-Specific Enhancement of Quality of Life Among Women With Breast Cancer: Systematic Review and Exploratory Meta-Analysis of Randomized Controlled Trials | No sufficient data (forest plot) for re-analysis. |
| Pan et al (2017) | Could yoga practice improve treatment-related side effects and quality of life for women with breast cancer? A systematic review and meta-analysis | No sufficient data (sample size, mean, and SD) for re-analysis. |
| O’Neil et al (2020) | The Effect of Yoga Interventions on Cancer-Related Fatigue and Quality of Life for Women with Breast Cancer: A Systematic Review and Meta-Analysis of Randomized Controlled Trials | No sufficient data (sample size, mean, and SD) for re-analysis. |
| Morilla et al (2022) | The Pilates Method as an alternative approach to recovery in women with breast cancer: A systematic review | Spanish article, not in English. |
| Lee et al (2010) | Tai chi for breast cancer patients: A systematic review | Another larger meta-analysis studying the same outcome was chosen as the eligible meta-analysis |
| Cramer et al (2012) | Yoga for breast cancer patients and survivors: A systematic review and meta-analysis | Another larger meta-analysis studying the same outcome was chosen as the eligible meta-analysis |
| Zhang et al (2012) | Effects of yoga on psychologic function and quality of life in women with breast cancer: A meta-analysis of randomized controlled trials | Another larger meta-analysis studying the same outcome was chosen as the eligible meta-analysis |
| Yan et al (2014) | Lack of efficacy of Tai Chi in improving quality of life in breast cancer survivors: a systematic review and meta-analysis | Another larger meta-analysis studying the same outcome was chosen as the eligible meta-analysis |
| Pan et al (2015) | Tai Chi Chuan exercise for patients with breast cancer: A systematic review and meta-analysis | Another larger meta-analysis studying the same outcome was chosen as the eligible meta-analysis |
| Luo et al (2020) | Effect of Tai Chi Chuan in breast cancer patients: A systematic review and meta-analysis | Another larger meta-analysis studying the same outcome was chosen as the eligible meta-analysis |
| Hsueh et al (2021) | Effects of yoga on improving quality of life in patients with breast cancer: a meta-analysis of randomized controlled trials | Another larger meta-analysis studying the same outcome was chosen as the eligible meta-analysis |
| Yi et al (2021) | Effects of yoga on health-related quality, physical health and psychological health in women with breast cancer receiving chemotherapy: a systematic review and meta-analysis | Another larger meta-analysis studying the same outcome was chosen as the eligible meta-analysis |
| Gong et al (2022) | Baduanjin exercise for patients with breast cancer: A systematic review and meta-analysis | Another larger meta-analysis studying the same outcome was chosen as the eligible meta-analysis |
| Hou et al (2024) | Effect of yoga on cancer-related fatigue in patients with breast cancer: A systematic review and meta-analysis | Another larger meta-analysis studying the same outcome was chosen as the eligible meta-analysis |

# Table S3. Characteristics of included meta-analyses.

| **First author (year)** | **Search time, included databases** | **Type of review** | **No. of study** | **Sample size, study population** | **Intervention/**  **Control^†^** | **Interest of outcomes** | **Outcome measurements** | **Quality assessment of included studies** |
| --- | --- | --- | --- | --- | --- | --- | --- | --- |
| Cramer  (2017) | From inception to Jan 29^th^ 2016, Cochrane Breast Cancer Specialised Register, Medline, Embase, Cochrane Central Register of Controlled Trials, WHO International Clinical Trials Registry Platform, Clinicaltrials.gov, Indexing of Indian Medical Journals | MA of RCTs | 24 | 2166, women with a diagnosis of non-metastatic or metastatic BC (at any stage) | Yoga/any type of control | HRQoL,  Anxiety | - FACT-B, SF-36, FACT-G, Lymphoedema QoL, EORTC QLQ-C30, FLI-C, EORTC QLQ-BR23; - HADS, STAI, DMI, PMS-SF | RoB1^‡^:  a: 17/7 trials for low/unclear ROB  b: 14/9/1 trials for low/unclear/high ROB  c: 1/5/18 trials for low/unclear/high ROB  d: 5/18/1 trials for low/unclear/high ROB  e: 15/4/5 trials for low/unclear/high ROB  f: 18/2/4 trials for low/unclear/high ROB  g: 18/2/4 trials for low/unclear/high ROB |
| Dong  (2019) | From inception to Jan 2019, Cochrane Central Register of Controlled Trials, PubMed, Ovid-Medline, Web of Science, China Biology Medicine, VIP, CNKI, Wanfang Data | MA of RCTs | 17 | 2183, adult patients diagnosed with BC, regardless of cancer stage and current treatment | Yoga/any type of control | CRF | - EORTC QLQ-C30, FACIT-F, FSI, Fatigue Likert Scale, VAS, CFS, FSS, CFS-D, MFSI-SF, BFI, FQL, MFI | RoB1^‡^:  a: 9/7/1 trials for low/unclear/high ROB  b: 7/10 trials for low/unclear ROB  c: 17 trials for high ROB  d: 2/14/1 trials for low/unclear/high ROB  e: 13/4 trials for low/high ROB  f: 17 trials for low ROB  g: 14/1/2 trials for low/unclear/high ROB |
| Li  (2023) | From inception to Sep 30^th^ 2022, PubMed, Web of Science, Cochrane Library, Embase, CNKI, Sinomed, VIP, Wanfang Data | MA of RCTs | 15 | 1156, adult female patients diagnosed as stage 0-III of primary BC | Tai Chi Chuan/any type of control | HRQoL, CRF,  Sleep quality | - WHOQOL-BREF, FACT-B, FACIT-F - FSI, CFS, PFS-R - PSQI | RoB1^‡^:  a: 12/3 trials for low/unclear ROB  b: 6/9 trials for low/unclear ROB  c: 1/14 trials for low/high ROB  d: 6/8/1 trials for low/unclear/high ROB  e: 12/3 trials for low/high ROB  f: 13/2 trials for unclear/high ROB  g: 15 trials for low ROB |
| Liu (2020) | From inception to June 2019, Ovid Medline, Amed, Embase, CINAHL, Web of Science, Cochrane Central Register of Controlled Trails, CNKI, Wanfang Data | MA of RCTs | 16 | 1268, adult patients diagnosed with breast cancer who received active breast cancer treatment | Tai Chi Chuan/any type of control | Depression | IDS, BDI, CES-D | PEDro:  14/2 trials for moderate to high/low quality  Mean score 6.56 (total score 11) |
| Liu (2021) | From inception to Jan 6^th^ 2021, PubMed, Web of Science, Cochrane Library, Embase, CNKI, Wanfang Data, SinoMed | MA of RCTs | 16 | 1133, adult patients diagnosed with both BC and cancer-related fatigue | Mind-body exercises/any type of control | CRF | BFI, EORTC QLQ-C30, CFS, FACIT-F, FSI, PFS-R | RoB1^‡^:  a: 10/4/2 trials for low/unclear/high ROB  b: 7/7/2 trials for low/unclear/high ROB  c: 2/13/1 trials for low/unclear/high ROB  d: 2/14 trials for low/unclear ROB  e: 12/1/3 trials for low/unclear/high ROB  f: 16 trials for low ROB  g: 16 trials for unclear ROB |
| Meng (2021) | From inception to Mar 2020, PubMed, Web of Science, Cochrane Central Register of Controlled Trails, CNKI, Wanfang Data, Sinomed | MA of RCTs, non-RCTs | 17 (14RCTs, 3nRCTs) | 1236 (1082 of RCTs), adult patients diagnosed with BC, regardless of BC stage and previous or current treatment | Qigong (subgroup: Baduanjin)/any type of control | HRQoL,  CRF,  Sleep quality,  Depression | - FACT-G, SF-36, FACT-B, EORTC QLQ-C30; - BFI, PFS, Fatigue score, Symptom score; - PSQI, Sleep disturbance score, - CES-D, BDI, SDS, HADS, PHO | 12 CBRG criteria:  Mean score 4.18 (total score 12) |
| Ye  (2022) | From inception to Dec 15^th^ 2021, PubMed, Web of Science, Cochrane Library, CNKI, VIP, Wanfang Data, CBMDisc | MA of RCTs | 7 | 450, postoperative patients with BC, regardless of nationality, race, and age | Baduanjin/any type of control | Anxiety | - SAS | RoB1^‡^:  a: 5/2 trials for low/unclear ROB  b: 3/4 trials for low/unclear ROB  c: 2/5 trials for low/unclear ROB  d: 2/5 trials for low/unclear ROB  e: 5/2 trials for low/unclear ROB  f: 7 trials for low ROB  g: 6/1 trials for low/unclear ROB |
| Zhu (2023) | From inception to June 8^th^ 2022, PubMed, Embase, CINAHL, Cochrane Library, Web of Science | MA of RCTs | 12 | 782, adult patients with a pathological diagnosis of BC | Yoga/any type of control | Sleep quality | PSQI, MOS-SS, EORTC QLQ C30-insomnia, Frequency of sleep disorders, Sleep quality VAS | RoB1^‡^:  a: 12 trials for low ROB  b: 6/6 trials for low/unclear ROB  c: 4/8 trials for low/high ROB  d: 12 trials for low ROB  e: 8/4 trials for low/unclear ROB  f: 12 trials for low ROB  g: 12 trials for unclear ROB |
| Zuo (2016) | From inception to Oct 2015, PubMed, Elsevier, Web of Science, Cochrane Library, CNKI, CBM, VIP, Wanfang Data | MA of RCTs | 21 | 1762, adult patients with BC | Yoga/any type of control | Depression | HADS, BDI, CES-D, SDS | RoB1:  detailed results for each study were not reported |

Notes: “^†^”: any type of control including usual care, wait-list control, psychotherapy, or other active exercise placebo control; “^‡^”: RoB1 criteria including a. random sequence generation; b. allocation concealment; c. blinding of participants and personnel; d. blinding of outcome assessment; e. incomplete outcome data; f. selective reporting; g. other biases;

Abbreviations: BC: breast cancer; BDI: Beck Depression Inventory; BFI: Brief Fatigue Inventory; CES-D: Center for Epidemiological Studies Depression scale; CFS: Cancer Fatigue Scale; CFS-D: 15-item Cancer Fatigue Scale; CRF: cancer-related fatigue; DMI: Distressed Mood Index; EORTC QLQ-BR23: European Organization for the Research and Treatment of Cancer QLQ-Breast23; EORTC QLQ-C30: European Organization for the Research and Treatment of Cancer QLQ-C30; EORTC QLQ C30-insomnia: European Organization for the Research and Treatment of Cancer QLQ-insomnia; FACIT-F: Functional Assessment of Chronic Illness Therapy- Fatigue; FACT-B: Functional Assessment of Cancer Therapy- Breast; FACT-G: Functional Assessment of Cancer Therapy- General; FLI-C: Functional Living Index-Cancer; FQL: Fatigue Quality List; FSI: Fatigue Symptom Inventory; FSS: Fatigue Severity Scale; HADS: Hospital Anxiety and Depression Scale; HRQoL: health-related quality of life; IDS: Inventory of Depressive Symptoms; MA: meta-analysis; MFI: Multidimensional Fatigue Inventory; MFSI-SF: Multidimensional Fatigue Symptom Inventory-Short Form; MOS-SS: Medical Outcomes Study- Sleep Scale;nRCTs; non-randomized controlled trials; PEDro: physiotherapy evidence databases (PEDro) scale; PFS: Piper Fatigue Scale; PFS-R: Revised Piper Fatigue Scale; PHO: Patient Health Questionnaire; PMS-SF: Profile of Mood States-Short Form; PSQI: Pittsburgh Sleep Quality Index; RCTs: randomized controlled trials; SAS: Self-rating Anxiety Scale; SF-36: Medical Outcomes Study 36-item Short Form Survey; SDS: Self-rating Depression Scale; Sleep quality VAS: Sleep Quality Visual Analogue Score; STAI: State Trait Anxiety Inventory; VAS: Visual Analogue Scale; WHOQOL-BREF: World Health Organization quality of life brief questionnaire;12 CBRG: 12 Cochrane back review group for risk of bias.

# Table S4. AMSTAR-2 rating.

| **Reference**  **(First author, year)** | **AMSTAR-2 Item** | | | | | | | | | | | | | | | | **Level** |
| --- | --- | --- | --- | --- | --- | --- | --- | --- | --- | --- | --- | --- | --- | --- | --- | --- | --- |
|  | **Q1** | **Q2** | **Q3** | **Q4** | **Q5** | **Q6** | **Q7** | **Q8** | **Q9** | **Q10** | **Q11** | **Q12** | **Q13** | **Q14** | **Q15** | **Q16** |  |
| Cramer, 2017 | Yes | No | Yes | Yes | Yes | Yes | Yes | Yes | Yes | No | Yes | Yes | Yes | Yes | Yes | Yes | Low |
| Dong, 2019 | Yes | No | Yes | Yes | Yes | Yes | Yes | Partial Yes | Yes | No | Yes | Yes | Yes | Yes | Yes | Yes | Low |
| Li, 2023 | Yes | Yes | Yes | Yes | Yes | Yes | Yes | Partial Yes | Yes | No | Yes | Yes | Yes | Yes | No | Yes | Low |
| Liu, 2020 | Yes | No | Yes | Yes | Yes | Yes | Partial Yes | Yes | Yes | No | Yes | Yes | No | Yes | No | Yes | Critically Low |
| Liu, 2021 | Yes | Yes | Yes | Yes | Yes | Yes | Partial Yes | Partial Yes | Yes | No | Yes | Yes | No | Yes | No | Yes | Critically Low |
| Meng, 2018 | Yes | No | Yes | Yes | Yes | No | Partial Yes | Yes | Yes | No | Yes | Yes | Yes | Yes | Yes | Yes | Critically Low |
| Ye, 2022 | Yes | Yes | Yes | Yes | Yes | Yes | Partial Yes | Partial Yes | Yes | No | Yes | No | No | Yes | No | Yes | Critically Low |
| Zhu, 2023 | Yes | No | Yes | Yes | Yes | Yes | Partial Yes | Yes | Yes | No | Yes | Yes | Yes | Yes | Yes | Yes | Critically Low |
| Zuo, 2016 | Yes | No | Yes | Yes | Yes | Yes | No | Partial Yes | Yes | No | Yes | Yes | No | Yes | Yes | Yes | Critically Low |

Q = Question; AMSTAR 2 critical domains included Q2, Q4, Q7, Q9, Q11, Q13, and Q15

# Table S5. Quantitative synthesis and evidence grading criteria of the 16 meta-analyses comparing mind-body exercise interventions and controls.

| **Outcome** | **Intervention** | **Source** | **No. of study /BC cases estimates** | **No. of BC cases (I/C)** | **Random effect size (95% CI)**  **and *P* value**^†^ | **I^2^(%)** | **95%PI** | **Largest study 95%CI** | **Egger**  ***P* value** | **Excess significance bias** | | | **AMSTAR2**  **grading** |
| --- | --- | --- | --- | --- | --- | --- | --- | --- | --- | --- | --- | --- | --- |
|  |  |  |  |  |  |  |  |  |  | **O** | **E** | **P value** |  |
| **Convincing (Class Ⅰ)** | | | | | | | | | | | | | |
| Depression | Yoga | Zuo,  2016 | 13/1150 | 565/585 | -0.77 (-0.93, -0.61)  P = 1.1×10^-20^ | 39.7 | -1.21 to -0.33 | -1.01 to -0.44 | 0.94 | 11 | 10.82 | 0.62 | CL |
| **Weak (Class Ⅳ)** | | | | | | | | | | | | | |
| Depression | Qigong | Meng,  2021 | 6/540 | 276/264 | -0.32 (-0.59, -0.04)  P = 0.023 | 58.8 | -1.13 to 0.50 | -0.36 to 0.27 | 0.07 | 2 | 0.33 | 0.039 | CL |
| HRQoL | Yoga | Cramer, 2017 | 15/1018 | 523/495 | 0.24 (0.04, 0.43)  P = 0.019 | 58.4 | -0.43 to 0.90 | 0.08 to 0.66 | 0.64 | 3 | 5.20 | 0.93 | L |
| HRQoL | Tai Chi Chuan | Li,  2023 | 6/402 | 201/201 | 0.35 (0.15, 0.54)  P = 6.1×10^-4^ | 0 | 0.07 to 0.63 | -0.01 to 0.84 | 0.46 | 1 | 1.81 | 0.88 | L |
| HRQoL | Qigong | Meng,  2021 | 11/832 | 413/419 | 0.58 (0.14, 1.02)  P = 0.011 | 89.2 | -1.08 to 2.24 | -0.28 to 0.34 | 0.08 | 6 | 0.57 | 7.4×10^-6^ | CL |
| HRQoL | Baduanjin | Meng,  2021 | 6/421 | 213/208 | 0.98 (0.26, 1.70)  P = 0.0075 | 91.6 | -1.61 to 3.58 | -0.31 to 0.54 | 0.01 | 5 | 0.46 | 1.5×10^-5^ | CL |
| CRF | Yoga | Dong,  2019 | 17/984 | 514/470 | -0.35 (-0.60, -0.10)  P = 0.0060 | 75.7 | -1.39 to 0.69 | -0.30 to 0.38 | 0.07 | 7 | 1.06 | 5.0×10^-5^ | L |
| CRF | Tai Chi Chuan | Li,  2023 | 4/247 | 125/122 | -0.85 (-1.47, -0.23)  P = 0.0071 | 80.2 | -3.62 to 1.92 | -0.53 to 0.31 | 0.28 | 3 | 0.28 | 0.0014 | L |
| CRF | Mind-body exercises | Liu,  2021 | 16/1091 | 529/562 | 0.52^‡^ (0.15, 0.90)  P = 0.0059 | 88.1 | -1.04 to 2.08 | -0.18 to 0.44 | 0.32 | 8 | 1.32 | 1.5×10^-5^ | CL |
| Anxiety | Yoga | Cramer, 2017 | 9/541 | 295/246 | -1.10 (-1.79, -0.42)  P = 0.0017 | 92.1 | -3.60 to 1.39 | -0.65 to 0.09 | 0.05 | 4 | 1.67 | 0.068 | L |
| Anxiety | Baduanjin | Ye,  2022 | 3/191 | 96/95 | -1.56 (-2.62, -0.50)  P = 0.0040 | 90.2 | -14.79 to 11.68 | -1.27 to -0.23 | 0.01 | 3 | 3 | 1 | CL |
| Sleep quality | Yoga | Zhu,  2023 | 12/789 | 393/396 | -0.40 (-0.71, -0.09)  P = 0.011 | 76.0 | -1.48 to 0.68 | -0.53 to 0.14 | 0.05 | 4 | 1.46 | 0.049 | CL |
| **Not significant (Class NS)** | | | | | | | | | | | | | |
| Depression | Tai Chi Chuan | Liu,  2020 | 3/211 | 108/103 | 0.22 (-0.05, 0.49)  P = 0.12 | 0 | -1.55 to 1.99 | -0.30 to 0.53 | 0.58 | 0 | 0.23 | 1 | CL |
| CRF | Qigong | Meng,  2021 | 6/401 | 195/206 | -0.32 (-0.71, 0.07)  P = 0.11 | 72.6 | -1.59 to 0.96 | -0.24 to 0.57 | 0.42 | 2 | 0.61 | 0.12 | CL |
| Sleep quality | Tai Chi Chuan | Li,  2023 | 3/253 | 125/128 | -0.17 (-0.54, 0.20)  P = 0.37 | 55.3 | -4.10 to 3.76 | -0.76 to 0.09 | 0.17 | 0 | 1.01 | 1 | L |
| Sleep quality | Qigong | Meng,  2021 | 4/398 | 240/158 | -0.11 (-0.74, 0.52)  P = 0.73 | 85.8 | -3.01 to 2.79 | -0.24 to 0.57 | 0.55 | 1 | 0.44 | 0.37 | CL |

Note: “^†^”: all estimates in the original meta-analysis were converted to Hedges’ G to facilitate interpretation; “^‡^”: this study calculated mean difference with this formula “M = M1(pre) – M2(post)”, the results indicated cancer-related fatigue symptom reduction.

Abbreviations: “HRQoL”: health-related quality of life; CRF: cancer-related fatigue”; “I/C”: Intervention/Control; “CI”: confidence interval; “PI”: prediction interval; “O”: the observed number of studies; “E”: the expected number of significant results; “L”: low; “CL”: critically low
